# Bacterial growth and antimicrobial resistance in urinary *Escherichia coli* isolates among men with lower UTI in Swedish primary healthcare: retrospective data over a 4 year period

**DOI:** 10.1093/jacamr/dlae214

**Published:** 2024-12-26

**Authors:** Helena Kornfält Isberg, Martin Sundqvist, Eva Melander, Anders Beckman, Katarina Hedin

**Affiliations:** Department of Clinical Sciences, Family Medicine Malmö, Lund University, Malmö, Sweden; Department of Laboratory Medicine, Clinical Microbiology, Faculty of Medicine and Health, Örebro University, Örebro, Sweden; Regional Center for Communicable Disease Control, Region Skåne, Malmö, Sweden; Department of Translational Medicine, Lund University, Malmö, Sweden; Department of Clinical Sciences, Family Medicine Malmö, Lund University, Malmö, Sweden; Department of Clinical Sciences, Family Medicine Malmö, Lund University, Malmö, Sweden; Futurum, Region Jönköping County and Department of Health, Medicine and Caring Sciences, Linköping University, Linköping, Sweden

## Abstract

**Background:**

*Escherichia coli*, the most common bacterium causing urinary tract infections (UTIs), is increasingly reported as resistant to multiple antibiotics. Swedish surveillance data from hospital and primary health care (PHC) report a 17%–19% prevalence of resistance to ciprofloxacin in *E. coli* from urine cultures in men over 20 years of age. Surveillance data may include nosocomial infections. However, few studies have described resistance in *E. coli* in men with community-acquired UTI in PHC. We aimed to describe the microbiological results, including antibiotic resistance in *E. coli*, in men with lower UTI (LUTI) attending PHC.

**Methods:**

In this retrospective study based on information from electronic medical records, we included patients from 289 PHC centres. For all men aged 18–79 years diagnosed with LUTI in PHC from January 2012 to December 2015, we extracted data on age, UTI diagnosis and results from urine cultures.

**Results:**

A total of 17 987 episodes of lower UTI were identified. *E. coli* was detected in 62% of positive cultures and 63% of detected *E. coli* isolates were susceptible to all tested antimicrobials. Resistance in *E. coli* to the first-choice antibiotics pivmecillinam and nitrofurantoin were 2% and 1%, respectively. Resistance to ciprofloxacin was 9%, and to trimethoprim it was 17%.

**Conclusions:**

Resistance levels for ciprofloxacin in *E. coli* among men with LUTI in PHC were lower than in surveillance data. The results of this study point to the importance of surveillance of resistance in urine samples from patients with LUTI in PHC in order to choose the right empirical antibiotic treatment.

## Introduction

According to the WHO, increasing antimicrobial resistance (AMR) is 1 of 10 threats to global health. To fight this, the WHO adopted a global action plan on AMR in 2015. One of the five objectives of the plan was to strengthen the knowledge and evidence base through surveillance and research. The importance of examining AMR patterns in terms of gender and other relevant social stratifiers is emphasized.^1^

*Escherichia coli* is the most common bacterium causing urinary tract infections (UTIs) and is increasingly reported worldwide as resistant to multiple relevant antibiotics.^2^ In Sweden, AMR data from all clinical isolates from most of the clinical microbiological laboratories is collected by an automated system (SVEBAR) and compiled in a national yearly report—*Swedres*. Data reported in 2015 and 2016^3^ showed that *E. coli* was the most common pathogen in culture-positive urine samples from men (41%). The resistance to fluoroquinolones in *E. coli* was 10%, with both men and women included. In 2020, the ciprofloxacin resistance level in men over 20 years of age was 17%–19%.^4^ Few recent studies have described antibiotic resistance in *E. coli* in men with community-acquired UTI in primary health care (PHC). In a study from the Netherlands from 2009 to 2011, *E. coli* accounted for 51% of the total isolates in urine cultures from men with UTI in PHC and antibiotic susceptibility was lower among men with UTI compared with women.^5^

The incidence of lower UTI (LUTI) in men varies between countries and age groups and is 0.9–16 per 1000 men per year.^5–7^ LUTI, defined as non-febrile UTI not affecting the upper urinary tract in men, is in many cases diagnosed and treated in PHC in Sweden.^3^

Antibiotic resistance surveillance based on routine cultures is an important tool for following resistance development. However, these data need to be further analysed on the species level and with a focus on sex and age groups to provide detailed information to be optimally used for recommendations for first-line treatment. In the current study, our aim was to describe the microbiological results, including antibiotic resistance, in men with LUTI attending PHC. We also aimed to describe the susceptibility pattern in *E. coli* in relation to clinical outcome.

## Materials and methods

This study was based on a previous study describing outcomes in relation to antibiotic treatment in men with LUTI.^8^ In this study, we only included patients with index visits in PHC. We analysed reports regarding bacterial growth and AMR from clinical urine cultures collected from male patients diagnosed with LUTI, which were all handled in PHC. The results from the urine cultures were also described in relation to treatment outcome regarding therapy failure, recurrence and complications. The same definitions of the UTI, index visit, antibiotic use, study antibiotic, treatment duration, therapy failure, recurrence and complications were used as in the previous study.^8^

We included 289 PHC centres (PHCCs) in five counties in Sweden (Jönköping, Kalmar, Kronoberg, Skåne and Uppsala). Register data on all men aged 18–79 years diagnosed with LUTI or complications pertaining to UTI in PHC and hospital care from January 2012 to December 2015 were extracted from electronic medical records. Data from hospital care were extracted in order to find information on urine cultures from patients who later sought hospital care due to complications. Due to technical problems with data management, data from Skåne county were collected from December 2012 to December 2015. We extracted data on age, UTI diagnosis, day of consultation, prescribed antibiotic and results from urine culture collected within 5 days from the consultation in PHC. Methods and design are described in detail in the previously published study.^8^

Patients with an ICD code indicating LUTI and an adjacent antibiotic prescription within 5 days of the date of the registered index diagnosis were included.

To separate index visits from follow-up visits, we excluded patients with UTI diagnoses with an antibiotic prescription in the preceding 30 days. Visits during the first 30 days of the study were excluded since there was no information on antibiotic prescribing for the preceding 30 days. Visits were included until 31 December 2015.

The follow-up period after an index visit was at most 35 days. The same patient could be included more than once in the study.

### Bacteria and antibiotic susceptibilities

Culture and susceptibility testing had been performed at five different laboratories (the Departments of Clinical Microbiology at the university hospitals of Uppsala and Lund, and the regional hospitals in Jönköping, Växjö and Kalmar); all used accredited methods for urinary culture and susceptibility testing during the study period.^9^

Bacterial cultures and species determination were performed using standard methods at the laboratories at the time of the study. Cultures were considered positive for *E. coli* and *Staphylococcus saprophyticus* in the case of bacterial growth of ≥10^3^ cfu/mL. For other pathogens, the limit of ≥10^4^ cfu/mL was used. Three or more species were reported as mixed cultures and considered as contaminants. Urine cultures with no growth, mixed bacterial flora, skin flora or *Candida* were considered negative.

The antibiotic susceptibility was determined according to the disc diffusion method and followed guidelines and breakpoints proposed by EUCAST at the time of testing.^10^

Standard antibiotics for primary susceptibility testing were ampicillin, mecillinam, cefadroxil, trimethoprim, nitrofurantoin and ciprofloxacin. Pivmecillinam is the prodrug to mecillinam. Trimethoprim is used in the primary panel of susceptibility testing in UTI as it gives a very good prediction of susceptibility also to trimethoprim/sulfamethoxazole.

### Definitions

#### Index visit

A visit to PHC with a registered prescription of UTI relevant antibiotics on the day of visit or within 5 days.

#### Therapy failure

A new prescription of a UTI antibiotic and a new registered lower UTI diagnosis within 7 days from the index antibiotic prescription.

#### Recurrence

A new visit, a LUTI diagnosis and an antibiotic prescription 8 to 30 days from prior antibiotic prescription due to a UTI diagnosis.

#### Complication

A new visit to PHC or hospital care within 30 days from the index antibiotic prescription, combined with a diagnosis of pyelonephritis or sepsis, and combined with an antibiotic prescription registered on the day of the new visit.

### Statistical analysis

The data collected in the study were analysed using MATLAB 2019a (MathWorks, Natick, MA, USA) and Excel 2016 (Microsoft Corp., Redmond, WA, USA).

Data were mainly descriptive, with numbers and frequencies presented in tables. Descriptive statistics (median, IQR, mean, SD, percentages) were used to describe bacterial findings and antibiotic resistance in *E. coli.*

Comparisons between proportions of categorical variables in two independent groups were performed using the multiple-groups chi-squared test. Statistical significance was set at *P* < 0.05.

## Ethics

Confidentiality for patients was ensured by using an encrypted ID number. The study was approved by the Regional Ethical review Board in Lund, Sweden (Dnr 2016/462).

## Results

A total of 17 987 diagnoses that met the inclusion criteria for an index LUTI in 14 523 men diagnosed in PHC were identified. The median age based on the 17 987 diagnoses was 64 (IQR = 49–72) years. (Figure S1, available as Supplementary data at *JAC-AMR* Online). Among the 14 523 men, 84% (12 130/14 523 patients) had a single diagnosis of LUTI, 12% (1759/14 523 patients) had two diagnoses and 4% (634/14 523 patients) had three or more diagnoses during the four observed years.

### Patients and culture results

A urine culture was performed in 52% (9366/17 987) of diagnoses, and bacterial growth was reported in 54% (5064/9366) of these. Among the 5064 positive cultures, growth of a single bacterium was reported in 83% (4211/5064) of the samples and growth of two different species in 13% (669/5064). The most frequent bacterium found was *E. coli*, in 62% (3 152/5 064) of the positive cultures, followed by *Enterococcus* spp., in 12% (587/5064) and *Klebsiella pneumoniae* in 6% (291/5064) (Table 1).

**Table 1.** Distribution of bacterial species in urine cultures from men

Organism	Prevalence of organism (proportion of positive cultures *n* (%)
*E. coli*	3152 (62)
*Enterococcus* spp.	587 (12)
* Enterococcus faecalis*	574 (11)
* Enterococcus faecium*	13 (0.3)
*K. pneumoniae*	291(6)
*Staphylococcus aureus*	229 (5)
*Proteus mirabilis*	200 (4.1)
*Klebsiella oxytoca*	197 (4)
*Enterobacter* spp.	155 (3)
*Citrobacter* spp.	147 (3)
CoNS	126 (2)
*Pseudomonas aeruginosa*	111 (2)
*S. saprophyticus*	51 (1)
*Streptococcus agalactiae* (Group B streptococci)	26 (0.5)
Total number of positive cultures^a^	5064

Urine cultures were collected in relation to an index visit with a registered lower UTI diagnosis in primary care.

^a^More than one microorganism could be detected in one culture. The total number of positive findings per species is thus higher than the total number of positive cultures.

Of all LUTI diagnoses, 60% (10 861/17 987) were registered among men aged 60 to 79 years (Table 2). Urine culture results were, across all age groups, registered in conjunction with around 50% of the index visits, while the proportion of positive urine cultures differed between age groups and was highest in the oldest age group (Table 2). The proportion of *E. coli* in positive cultures was highest in the youngest age group at 69% (347/500). Among men in the oldest age group, *Enterococcus* spp. became more common, although *E. coli* was still the most common bacterium in this group at 49% (1931/3970) (Table 2). No growth was registered in 4302/9366 (46%) cultures.

**Table 2.** Distribution of LUTI diagnoses, number of cultures, proportion of positive cultures and isolated uropathogens per age category

	18–39 years *n* (%)	40–59 years *n* (%)	60–79 years *n* (%)	*P* value^a^	Total all ages *n* (%)
Number of LUTI diagnoses (proportion of total number of LUTI diagnoses, denominator 17 987)	2647 (15)	4479 (25)	10 861 (60)		17 987 (100)
Number of cultures (proportion of total number of cultures, denominator 9366)	1319 (14)	2316 (25)	5731 (61)		9366 (100)
Number and proportion of positive cultures (number of positive cultures/total number of cultures)	455 (35)	1253 (54)	3356 (59)	<0.001	5064
Organism					
*E. coli*	347 (69)	874 (62)	1931 (49)	<0.001	3152
*Enterococcus* spp.	24 (2)	101 (7)	462 (12)	<0.001	587
*Enterococcus faecalis*	23 (5)	96 (7)	455 (11)	0.718	574
*K. pneumoniae*	30 (6)	71 (5)	190 (5)	0.718	291
*Staphylococcus aureus*	11 (2)	54 (4)	164 (4)	0.054	229
*Proteus mirabilis*	12 (2)	39 (3)	149 (4)	<0.05	200
*Klebsiella oxytoca*	7 (1)	43 (3)	147 (4)	<0.05	197
*Enterobacter* spp. (*cloacae*)	15 (5)	32 (2)	108 (3)	0.484	155
*Citrobacter* spp.	7 (1)	30 (2)	110 (3)	0.054	147
CoNS	2 (0.4)	14 (1)	110 (3)	<0.001	126
*Pseudomonas aeruginosa*	10 (0.4)	26 (2)	75 (2)	0.947	111
*S. saprophyticus*	9 (2)	16 (1)	26 (1)	<0.05	51
*Streptococcus agalactiae* (Group B streptococci)	3 (1)	23 (2)	43 (1)	0.140	69
Total number of presented bacteria	500	1419	3970		5624^b^

Urine cultures were collected in relation to an index visit with a registered lower UTI diagnosis in primary care. Proportions are stated for each age group, for which the nominator is the number of positive findings in each age group and the denominator is the number of respective bacteria in each age group.

^a^ *P* values calculated using multiple-groups chi-squared test. Comparison between age groups. Differences between age groups are considered significant if the *P* value is less than 0.05.

^b^Up to two microorganisms were reported in some cultures. The total number of findings is thus more than 5064.

### Prevalence of antibiotic resistance in E. coli

Data on susceptibility testing results were available for >90% of the *E. coli* isolates. The antibiotic resistance levels in *E. coli* are presented in Table 3. In total, 63% (1986/3152) of the isolates were susceptible to all tested antibiotics. Levels of resistance to pivmecillinam and nitrofurantoin were 2% (71/3152) and 1% (15/3152), respectively. Levels of resistance to trimethoprim and ciprofloxacin were 17% (497/3152) and 9% (271/3152) (Table 3), respectively. The overall resistance in *E. coli* did not differ between age groups and year (Tables S1 and S2).

**Table 3.** Antibiotic resistance in *E. coli* against antibiotics commonly used in PHC

Antibiotic	Number of *E. coli* tested	Resistance, number of samples *n* (%)
Ciprofloxacin	2887	271 (9)
Trimethoprim	2893	497 (17)
Mecillinam	2890	71 (2)
Nitrofurantoin	2892	15 (1)
Cefadroxil	2888	96 (3)
Ampicillin^a^	899	267 (30)

^a^Ampicillin was only tested in three regions, but for all isolates and therefore included.

### Antibiotic use

The most frequently prescribed antibiotics were fluoroquinolones, prescribed in 46% (8208/17 987) of all index diagnoses, followed by pivmecillinam in 27% (4815/17 987), nitrofurantoin in 17% (3014/17 987), trimethoprim in 4% (756/17 987) and trimethoprim/sulfamethoxazole in 2% (406/17 987).

In Table 4, antibiotic resistance in *E. coli* is described in relation to prescribed antibiotic at the time of culture. Two patients treated with nitrofurantoin had growth of *E. coli* with resistance to nitrofurantoin (2/449; 0.4%). Correspondingly, eight patients treated with pivmecillinam had mecillinam-resistant *E. coli* (8/809; 1%) and 41 patients treated with fluoroquinolones displayed *E. coli* with resistance to ciprofloxacin (41/1626; 2%) (Table 4).

**Table 4.** AMR in *E. coli* isolates, from men with LUTI diagnoses presented based on the antibiotic prescribed at diagnosis

	Antibiotic treatment				
Antibiotic tested	Pivmecillinam *N* = 809, *n* (%)	Nitrofurantoin *N* = 449, *n* (%)	Fluoroquinolone^a^ *N* = 1626, *n* (%)	Trimethoprim *N* = 111, *n* (%)	Trimethoprim/sulfamethoxazole *N* = 83, *n* (%)
Ciprofloxacin	99 (12)	78 (17)	41 (2)	14 (13)	17 (20)
Trimethoprim	124 (15)	88 (20)	244 (15)	13 (12)	8 (10)
Mecillinam	8 (1)	21 (5)	34 (2)	2 (2)	3 (4)
Nitrofurantoin	10 (1)	2 (0)	1 (0)	0 (0)	1 (1)
Cefadroxil	35 (4)	16 (4)	28 (2)	7 (6)	5 (6)

For the proportions, nominator is the number of isolates with resistance and denominator is the number of patients treated with the specific antibiotic.

^a^Fluoroquinolones: ciprofloxacin (1621); norfloxacin (4); moxifloxacin (1).

### Therapy failure, recurrence and complications

Therapy failure was registered in 152 (0.8%), recurrence in 1140 (6%) and complications in 96 (0.5%) of cases. Urine culture results at the time of treatment were available in 55% (84/152) of the patients in the therapy failure group, 52% (597/1140) in the recurrence group and 53% (51/96) in the complications group. In the group defined as therapy failure, 26% (22/84) of the patients had been prescribed an antibiotic that was not effective against the microorganism found due to the antibacterial spectrum of the antibiotic or AMR, 1 to 22 days before. The corresponding figures for the recurrence and complications groups were 7% (44/597) and 41% (21/51), respectively.

In Table S3, antibiotic resistance in *E. coli* is described in patients prescribed an antibiotic at the index diagnosis and presented in the three outcome groups: therapy failure, recurrence and complications.

In summary, 14% (81/578) of patients who experienced therapy failure, recurrence or complications had initially been prescribed a non-effective antibiotic based on the antibacterial spectrum and the antimicrobial susceptibility profile.

A description of the bacterial findings in urine samples collected at index visits for LUTI is presented for the three outcomes: therapy failure, recurrence and complications (Table S4). The most common microorganism in all outcome groups was *E. coli* at 55% (36/66), 64% (300/470) and 74% (31/42), respectively.

## Discussion

This register-based study included 17 987 registered diagnoses of LUTI in men in five Swedish counties from 2012 to 2015. The overall findings showed that urinary cultures had been obtained and analysed in 52% of the visits and that fluoroquinolones were the most frequently prescribed antibiotic. The most common bacterium in urine samples was *E. coli*, but in older men, other species like *Enterococcus* spp. were increasingly common. The prevalence of antibiotic resistance in *E. coli* to nitrofurantoin and pivmecillinam was low and resistance to ciprofloxacin was below 10%.

Few studies describing antibiotic susceptibility patterns in urine cultures from men with UTI diagnosed in PHC have previously been published. The three studies we could find all included fewer patients than our study (422, 560 and 610 men).^5,11,12^ The mean age of our population was 64 years, which is older than two of the previous studies (57 and 62.5 years),^11,12^ but younger compared with a study from the Netherlands (65 years).^5^ The proportion of *E. coli* in positive urine cultures was 62% in this study and 50%, 51% and 48% in the studies from France and the Netherlands.^5,11,12^ The study by Soudais *et al.*^11^ had a slightly different population as it also included patients with complicated UTIs. Antibiotic resistance to ciprofloxacin in *E. coli* was 9% compared with 3% and 6% in the two studies from the Netherlands.^5,12^ The discrepancies in susceptibility to ciprofloxacin could be explained by the increasing prevalence of resistance to ciprofloxacin in *E. coli* over time as the two Dutch studies were performed earlier than our study (2009–11 and 2002–04, respectively). Other reasons might be differences in the prescribing pattern of ciprofloxacin between the different countries or differences in included patient populations. No specific antimicrobial stewardship campaign took place at the time of our study.

Swedish surveillance data from both hospitals and PHC from 2022 reported that resistance to ciprofloxacin in *E. coli* from urine cultures in men over 20 years was 17%–19%.^4^ The corresponding figure in our study was 9%. This difference could be due to increasing resistance in *E. coli* but more likely that the prevalence of antibiotic resistance in urine cultures from men attending PHC with signs of LUTI is lower than the prevalence of antibiotic resistance in urine cultures included in the national surveillance data, where both clinical isolates from hospitals and PHC are included and are not presented separately. Men attending hospital care likely have more complicated illnesses and may have been exposed to several antibiotic courses due to preceding UTIs. In addition, clinicians might order repeated cultures from patients with complicated resistance patterns. This could lead to a higher prevalence of antibiotic resistance in national surveillance data as—compared with our study—no adjustment for repeated findings of the same species is done. Furthermore, in contrast to surveillance data, in our study only men with LUTI diagnoses from PHC were included and patients with an antibiotic prescription within the preceding 30 days were excluded. The difference in resistance levels in urine samples between hospital care and PHC for ciprofloxacin in *E. coli* was also found in an earlier study on UTI in women.^13^ The differences in resistance levels between different settings is important knowledge in an era of increasing resistance as valid treatment options otherwise can be discarded based on surveillance data. Further studies with a prospective study design are thus needed. The Swedish national database for AMR surveillance (SVEBAR) is under reconstruction and will, in the coming years, include the Swedish personal identification number associated with all reports, giving the opportunity to analyse clinical data and resistance data in a better way at the national level. We believe that the data reported in this study on antibiotic resistance in *E. coli* among men with lower UTI in PHC are representative at a national level.

The most frequently used antibiotics were fluoroquinolones (46%), which is in line with earlier studies from other countries where fluoroquinolones were prescribed in 70%, 33%, 36% and 65% of cases.^11,12,14,15^ This was in line with guidelines when the study sample was collected (2012–15) but is not in line with the current Swedish guidelines implemented in 2017. Guidelines for male LUTI vary between countries and the infection is, in many countries, considered as complicated. In Sweden and other Nordic countries, narrow-spectrum antibiotics are recommended as the first-choice treatment. The background is an increasing number of *E. coli* resistant to fluoroquinolones, trimethoprim and trimethoprim/sulfamethoxazole. Men with UTI and fever (complicated UTI) should, according to Swedish guidelines, be treated with broad-spectrum antibiotics that will reach therapeutic concentrations of antibiotics in the kidneys, blood, urine and the prostate.^16^ The Swedish national treatment guidelines for LUTI in men 18 years and older now recommend treatment with pivmecillinam or nitrofurantoin for 7 days as the first antibiotic choice, and a urine culture should always be performed when empirical treatment is initiated. Fluoroquinolones should only be prescribed when there are no other alternatives to prevent further resistance development and to avoid side effects.^3,16^ It would thus be interesting to perform a new study with a similar design to address whether antibiotic prescribing patterns have changed according to the new guidelines.

Surveillance data from *E. coli* in urine cultures show resistance rates to fluoroquinolones that exceed 20% in most European countries.^17^ This means that ciprofloxacin, the former first-line antibiotic in men with lower UTI in Sweden, might be an inappropriate antibiotic choice due to high levels of resistance, and other first-line antibiotics are probably more effective in this group. Interestingly, we noted a lower ciprofloxacin resistance level in the group of patients receiving ciprofloxacin than in other groups thus indicating a pre-existing knowledge among the prescribing physicians regarding the susceptibility pattern of the bacteria causing infection in the specific patient. Ciprofloxacin should be preserved for serious infections as treatment with ciprofloxacin contributes to the selection of drug-resistant organisms and to increased rates of drug-related adverse events.

To our knowledge, this study is the largest diagnosis-linked study on LUTI in men diagnosed in PHC that also contains results from urine cultures, including resistance patterns. UTI is rare among younger men; the incidence increases with age. Contact rates in PHC due to UTI were 232/1000 women and 37/1000 men in the Netherlands in 2014^18^ and in Sweden, visits related to UTI were six times more common in women than in men.^7^ This means that studies related to male UTI are difficult to perform in PHC and prospective clinical data from men with UTI diagnoses in PHC are hard to retrieve. Many PHCCs need to be included to cover a population that is large enough; it is therefore important to extract data from available sources as in the present study. Resistance patterns differ between men and women, between patients in hospital and PHC, and between different geographic areas.^5,19,20^ The information about antibiotic resistance in urine cultures from men with UTI is important when developing guidelines for empirical antibiotic treatment within this demographic.

A strength of the study is the methodology for urine cultures. Culture and susceptibility testing were performed at five different laboratories in different Swedish counties. All were performed according to accredited methods for urinary culture and susceptibility testing at the time of the study and susceptibility testing was performed in a large proportion of the isolates.^9,10^

A limitation of the study is the retrospective study design since the data are not the most recent and we do not have information about patients’ clinical symptoms and comorbidities. Comorbidities such as indwelling catheters, urinary tract obstructions and fragile patients in general could affect the choice of antibiotic treatment and the outcome of treatment. Even though treatment guidelines state that a urine culture shall be performed in a man with LUTI, only half of the patients with a LUTI diagnosis had a urine culture performed. It could be that GPs who examined men with LUTI were more prone to order a urine culture in patients who were more affected by their infection. This could contribute to the higher percentage of *E. coli* among positive cultures in this study compared with previously cited studies^5,11,12^ and also the choice of ciprofloxacin as treatment. We do not know how the GPs confirmed the diagnosis, if the patient had a symptomatic UTI or if the diagnosis was made in connection with a check-up culture after a UTI. However, we tried to avoid the latter by not including patients with an antibiotic prescription in the preceding 30 days due to a UTI diagnosis. Previous studies suggest that GPs perceive that a UTI in a man is difficult and complex to diagnose^21,22^ and that guidelines from different countries are not coherent.^23^ This issue could lead to over-diagnosing, and some individuals with a UTI diagnosis in this study could, for example, instead represent patients with asymptomatic bacteriuria and symptoms of other causes. In our study, the proportion of positive cultures in younger men was low, which could represent symptoms from sexually transmitted infections (STIs) instead of LUTI. We do not have information about diagnostic tests for STI in the studied cohort.

This study is based on data from 2012–15. This means that information on resistance levels in PHC probably has changed from that time and more recent data are desirable. The latest surveillance data from *Swedres* show that the ciprofloxacin resistance level in *E. coli* is 10% when combining men and women, and for men aged 65–84 years is almost 20%.^4^ Corresponding figures for all isolates (including both men and women) were around 6% in 2015.

We observed that almost half of the patients in each outcome group (therapy failure, recurrence and complications) did not have their urine cultured at index visit. This is a concern since the result of the urine culture could have provided the GP with an early signal of possible failure due to a pathogen or resistance pattern not in line with the given antibiotics. Furthermore, the culture results could help in the case of therapy failure.^24^ Swedish treatment guidelines state that a urine culture should be performed when UTI in a man is suspected. To our knowledge, this strategy has not been evaluated but could be important to follow up based on the discrepancy seen in our study.

Among patients where a urine culture was obtained and analysed, growth of *E. coli* was the most common finding and resistance to commonly used antibiotics (except for trimethoprim) was under 10%. Importantly, only 14% (81/578) of patients who were defined as a therapy failure, recurrence or complication had initially been prescribed a non-effective antibiotic based on the antimicrobial susceptibility profile, indicating that current antibiotic guidelines for men with LUTI from 2017 (emphasizing nitrofurantoin and pivmecillinam) seem appropriate as long as the resistance rates of these antibiotics continue to be low.

### Conclusions

*E. coli* is the most common bacterium in urine cultures from adult men in all age groups with a diagnosis of LUTI in Swedish PHC. In *E. coli*, resistance levels to antibiotics recommended for treatment of LUTI in men were low. Resistance levels to ciprofloxacin were not as high as in surveillance data, indicating surveillance bias likely skewed by nosocomial urinary isolates. This study thus provides important data to guide empirical treatment in PHC. However, the use of fluoroquinolones should still be avoided, if not necessary, due to their high prevalence of side effects and risk of AMR development.^25,26^

Although urine cultures were only performed in 52% of index diagnoses in this study, limiting the robustness of our microbiological surveillance, the results of this study point to the importance of surveillance of bacterial growth and resistance in urine samples from patients with LUTI in PHC. It is essential that results from men and women are reported separately in surveillance data to choose the right empirical antibiotic treatment. There is a need for more studies in this field with diagnosis-linked microbiological data from PHC and better national surveillance systems are therefore required.

## Supplementary Material

dlae214_Supplementary_Data

## Supplementary data

Figure S1 and Tables S1 to S4 are available as Supplementary data at *JAC-AMR* Online.
