# Supplementary material for: Bacterial growth and antimicrobial resistance in urinary Escherichia coli isolates among men with lower UTI in Swedish primary healthcare: retrospective data over a 4 year period
Source: JAC Antimicrob Resist. 2024 Dec 26;7(1):dlae214. doi: 10.1093/jacamr/dlae214 (PMC11670775; doi:10.1093/jacamr/dlae214)
Supplement: dlae214_Supplementary_Data [file dlae214_supplementary_data.docx]

Supplement


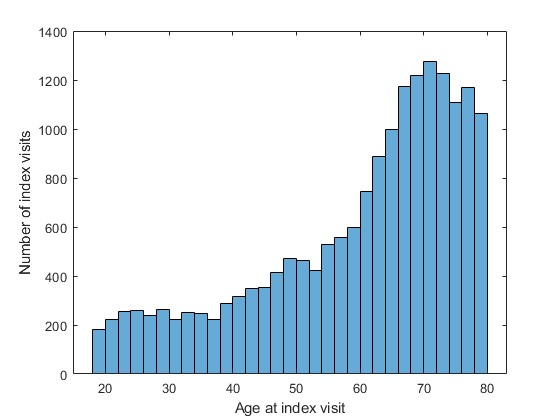


**Figure S1 Number of lower urinary tract infections episodes in men by different ages**

**Table S1 Proportion of resistant *E.coli* per age-group**

|  |  | **Age category (years)** | | | |
| --- | --- | --- | --- | --- | --- |
| Antibiotic tested | 18–39  (347) | 40–59  (875) | 60–80  (1932) | *p*-value* | Total all ages (3152) |
| Ciprofloxacin | 28 (8) | 58 (7) | 185 (10) | <0.05 | 271 (9) |
| Trimethoprim | 59 (17) | 139 (16) | 299 (15) | 0.77 | 497 (16) |
| Mecillinam | 9 (3) | 18 (2) | 44 (2) | 0.84 | 71 (2) |
| Nitrofurantoin | 2 (1) | 1 (0) | 12 (1) | 0.19 | 15 (1) |
| Cefadroxil | 11 (3) | 20 (2) | 65 (3) | 0.31 | 96 (3) |
| Ampicillin | 29 (8) | 73 (8) | 165 (9) | 0.98 | 267 (8) |

(Percentage within brackets with all samples with growth of *E.coli* in each age-group as the denominator)

* *p*-values calculated using multiple groups chi-2 test. Comparison between age groups. Differences between age groups are considered significant if the *p*-value is less than 0.05.

**Table S2 Antibiotic resistance in *Escherichia coli* against antibiotics commonly used in primary health care.**

| **Year** | 2012 | | 2013 | | 2014 | | 2015 | |
| --- | --- | --- | --- | --- | --- | --- | --- | --- |
| **Antibiotic** | No of *E.coli* tested | Resistance, No of samples  n (%) | No of *E.coli* tested | Resistance, No of samples  n (%) | No of *E.coli* tested | Resistance, No of samples  n (%) | No of *E.coli* tested | Resistance, No of samples  n (%) |
| Ciprofloxacin | 441 | 52 (12) | 638 | 71(11) | 933 | 84 (9) | 875 | 64 (7) |
| Trimethoprim | 445 | 80(18) | 638 | 108 (17) | 933 | 164(18) | 877 | 145(17) |
| Mecillinam | 446 | 10 (2) | 638 | 11(2) | 931 | 27(3) | 875 | 23(3) |
| Nitrofurantoin | 446 | 1 (0) | 638 | 3(0) | 932 | 4(0) | 876 | 7 (1) |
| Cephadroxil | 444 | 16 (4) | 637 | 16 (3) | 931 | 35(4) | 876 | 29(3) |
| Ampicillin* | 109 | 35 (32) | 127 | 30 (24) | 324 | 100(31) | 339 | 102(30) |

*Ampicillin was only tested in 3 regions, but on all isolates and therefore included

**Table S3 Therapy failure, recurrence and complication in relation to prescribed index antibiotic and bacterial findings in urine**

| **Antibioitc** | **Pivmecillinam**  (n=4815) | **Nitrofurantoin)** (n=3014) | **Ciprofloxacin** (n=8204) | **Trimethoprim** (n=756) | **Other antibiotic*** (n=792) |
| --- | --- | --- | --- | --- | --- |
| **Therapy failure (n=152)** | 59 | 37 | 43 | 8 | 2 |
| **Therapy failure, antibiotic resistance against prescribed antibiotic (n)** | Negative culture (n=7)No culture (n=32)  R Different species (n=1)  R Number of E.coli (n=0) | Negative culture (n=55)  No culture (n=21)  R Different species (n=3)  R Number of E.coli (n= 0) | Negative culture (n=6)  No culture (n=11)  R Different species (n=8)  R Number of E.coli (n=3) | Negative culture (n=1)  No culture (n=2)  R Different species (n=3)  R Number of E.coli (n=5) | Negative culture (n=0)  No culture (n=0 )  R Different species (n=0)  R Number of E.coli (n= 0) |
| **Recurrence (n= 1140)** | 462 | 258 | 287 | 52 | 48 |
| **Recurrence antibiotic resistance against prescribed antibiotic (n)** | Negative culture (n=42)  No culture (n=240)  R Different species (n=12)  R Number of E.coli (n=2) | Negative culture (n=23)  No culture (n=121)  R Different species (n=6)  R Number of E.coli (n=0) | Negative culture (n=50)  No culture (n=121)  R Different species (n=13)  R Number of E.coli (n=3) | Negative culture (n=4)  No culture (n=19)    R Different species (n=6)  R Number of E.coli (n=4) | Negative culture (n=7)  No culture (n=21)  R Different species (n=0)  R Number of E.coli (n=0) |
| **Complication (n=96)** | 23 | 18 | 40 | 3 | 9 |
| **Complication antibiotic resistance against prescribed antibiotic (n)** | Negative culture (n=2)  No culture (n=12)  R. Different species (n=3)  R Number of E.coli (n= 2) | Negative culture (n=0)  No culture (n=9)  R Different species (n=1)  R Number of E.coli (n=1) | Negative culture (n=7)  No culture (n=15)  R Different species (n=3)  R Number of E.coli (n=2) | Negative culture (n=0)  No culture (n=3)  R Different species (n=0)  R Number of E.coli (n=0) | Negative culture (n=0)  No culture (n=5)  R Different species (n=0)  R Number of E.coli (n=0) |

*Other antibiotic = (doxycycline, amoxicillin, amoxicillin/clavulanic acid, cefadroxil, ceftibuten, clindamycin**)**

**R Different species = Number of bacteria other than E. coli with resistance against the antibiotic in the table header**

**R Number of E. coli = Number of E. coli isolates with resistance against the antibiotic in the table header**

**Table S4. Distribution of bacterial species in positive urine cultures in men at index visit for LUTI in relation to outcome (therapy failure, recurrence and complication) ^*^**

| **Bacterial species** | **Therapy failure**  **n=66 (%)** | **Recurrence n=470 (%)** | **Complication n=42 (%)** |
| --- | --- | --- | --- |
| *Escherichia coli* | 36 (55) | 300 (64) | 31 (74) |
| *Enterococcus faecalis* | 13 (20) | 78 (17) | 3 (7) |
| *Klebsiella pneumoniae* | 4 (6) | 27 (6) | 4 (10) |
| *Staphylococcus aureus* | 3 (5) | 28 (6) | 2 (5) |
| *Koagulasnegativa stafylokocker* | 1 (2) | 16 (3) | 0 (0) |
| *Klebsiella oxytoca* | 4 (6) | 16 (3) | 0 (0) |
| *Proteus mirabilis)* | 4 (6) | 22 (5) | 1 (2) |
| *Pseudomonas aeruginosa* | 2 (3) | 16 (3) | 3 (7) |
| *Enterobacter spp.* | 2 (3) | 12 (3) | 1 (2) |
| *Staphylococcus saprophyticus* | 0 (0) | 5 (1) | 1 (2) |
| *Citrobacter spp.* | 5 (8) | 13 (3) | 1 (2) |
| *Streptococcus agalactiae (GBS)* | 2 (3) | 14 (3) | 1 (2) |
| Other*: Aerococcus,Acintobacter, Serratia, Providencia, Pantoea, Morganella Morgani* | 9 (14) | 21 (4) | 2 (2) |

*Growth of one or two different species in culture are reported. If more than two species are detected the culture is not reported (mixed growth).

Percentage within brackets: Nominator; number of positive cultures for each organism, denominator; total number of positive cultures in each outcome group.
